# Copper regulation of immune response and potential implications for treating orthopedic disorders

**DOI:** 10.3389/fmolb.2022.1065265

**Published:** 2022-12-05

**Authors:** Yamei Liu, Junlang Zhu, Liangliang Xu, Bin Wang, Weiping Lin, Yiwen Luo

**Affiliations:** ^1^ College of Basic Medical, Guangzhou University of Chinese Medicine, Guangzhou, China; ^2^ Research Center of Integrative Medicine, School of Basic Medical Sciences, Guangzhou University of Chinese Medicine, Guangzhou, China; ^3^ Department of Trauma and Foot-Ankle Surgery, The Second Affiliated Hospital of Guangzhou University of Chinese Medicine, Guangzhou, China; ^4^ Lingnan Medical Research Center, The First Affiliated Hospital of Guangzhou University of Chinese Medicine, Guangzhou University of Chinese Medicine, Guangzhou, China; ^5^ Department of Traumatology, The Third Affiliated Hospital of Guangzhou University of Chinese Medicine, Guangzhou, China; ^6^ The Fifth Affiliated Hospital of Guangzhou Medical University, Guangzhou, China

**Keywords:** copper, immune response, osteoporosis, arthritis, fracture, mesenchymal stem cells, cu-containing biomaterials

## Abstract

Copper is an indispensable trace metal element in human body, and copper deficiency is rare in clinic. However, diseases associated with serum copper deficiency, such as leukopenia, neutropenia, arthritis, osteoporosis, and bone defects, are well known. Copper ions can also achieve the effect of fighting pathogenic bacteria through the “contact killing” characteristic. Copper ion is also an important cofactor of bone matrix synthase, plays an important role in the pathophysiology of orthopedic diseases. The present review highlights the biological functions of copper in immunity, bone diseases and stem cells, as well as potential drug development targeting copper status for diagnostics and therapeutics of copper-associated bone diseases.

## Introduction

Copper, an essential trace element as important co-factors of various chaperones and enzymes, is vital for maintenance of integrity and homeostasis of the human organism, such as the skeletal system (Pavelková et al., 2018; Maung et al., 2021). This current review summarizes advancement of copper involvement of immune response, and potential clinical implication for management of orthopedic diseases.

## Copper regulation of the immune system

Trace element copper is necessary for a series of human physiological processes, including damage site repair and immune system function (Livingstone, 2017). Copper is an essential redox-active trace element for proper functioning of almost all organisms (Prohaska and Lukasewycz, 1990; Tapiero and Tew, 2003).

Generally, copper intake for infants of 0–6 months is 200, and 220 mg/d for 7–12-month-old infants. The intake of copper in children aged 1–3 years was 340 mg/day. Copper intake for children aged 4–8 was 440 mg/day; Copper intake for children aged 9–13 was 700 mg/day; Copper intake for adults 19–50 is 900 mg/day. 1,000 mg/day during pregnancy and 1,300 mg/day during lactation. Therefore, copper demand increases during pregnancy and lactation. Copper gluconate is the only copper supplement listed in the United States Pharmacopoeia Convention for oral use. Copper in food is mainly absorbed through the stomach and the upper part of the small intestine (Turnlund et al., 1997). Fifty years ago, Newberne et al. (1968) observed that copper-deficient mice infected with *Salmonella typhimurium* died faster and lived shorter than mice infected only with *S. typhimurium* in the control group.

Notably, copper is critical for immune functioning (Sullivan and Ochs, 1978; Prohaska and Lukasewycz, 1990; Tapiero and Tew, 2003). Copper metabolism changes during inflammation, and the level of serum copper metabolism increases. The lack of copper will lead to impaired energy production and affect the operation of the immune system (Hordyjewska et al., 2014). The immune system, mainly including innate and adaptive immunity, is a natural defensive response to stimulating factors such as infection, injury, and toxins (Medzhitov, 2008). Whilst excessive or improper immune response would cause numerous inflammatory diseases, such as cardiovascular disease (Ferrucci and Fabbri, 2018), and arthritis (Davidson and Diamond, 2001). Increasing evidence suggests the linking between copper deficiency and immune hypo-responsiveness (Immune tolerance). Reduced dietary copper after parturition causes immune suppression characterized by hypo-responsiveness to sheep red blood cells, B- and T-cell mitogens and alterations in splenic lymphocyte subpopulations (Prohaska and Lukasewycz, 1981; Lukasewycz and Prohaska, 1983; Vyas and Chandra, 1983; Lukasewycz et al., 1985; Blakley and Hamilton, 1987). Copper deficiency would cause the alteration of the acute-phase protein response to viral infection and may also affect lymphocyte responsiveness to mitogen stimulation (Arthington et al., 1996). During infection and inflammation, serum copper concentration increases, and ceruloplasmin activity also increases. These changes in copper metabolism result from interleukin-1-mediated increases in liver ceruloplasmin synthesis and release, an acute phase protein (Pekarek et al., 1972). Barber and Cousins showed that ceruloplasmin synthesis could be induced by intraperitoneal injection of interleukin-1 regardless of copper status (Barber and Cousins, 1988). Therefore, copper ion supplementation is necessary for ceruloplasmin synthesis in copper-deficient mice (Stabel and Spears, 1989). Thus, copper plays an important role in the mammalian immune system, and bacterial responses to copper could be a suitable target for future drug development.

The “contact killing” toxicity of copper alloy surface materials to clinically relevant pathogens can reduce the transmission of clinically relevant pathogens (Giachino and Waldron, 2020). In general, copper contributes to immunity through two pathways : 1) by participating in the development and differentiation of immune cells, and 2) by providing antifungal agents isolating machinery by metal in the host or as a bombarding property. Macrophages fight against invading pathogens by mobilizing copper ions in the body to create a copper-rich environment. The role of copper in the axis of infection has been further clarified by studies of copper excess or copper deficiency in resistance to pathogens such as *mycobacterium Escherichia coli* and Salmonella (Li et al., 2019). A large amount of evidence shows that copper regulation is key in innate and adaptive immune regulation. Disrupting key copper regulation often leads to a sharp increase in the pathogenicity of pathogens in the human body. In the future, copper containing complexes and body copper regulation will be the research hotspot of resistance to pathogens.

## Application of copper in bone diseases

**FIGURE 1:**
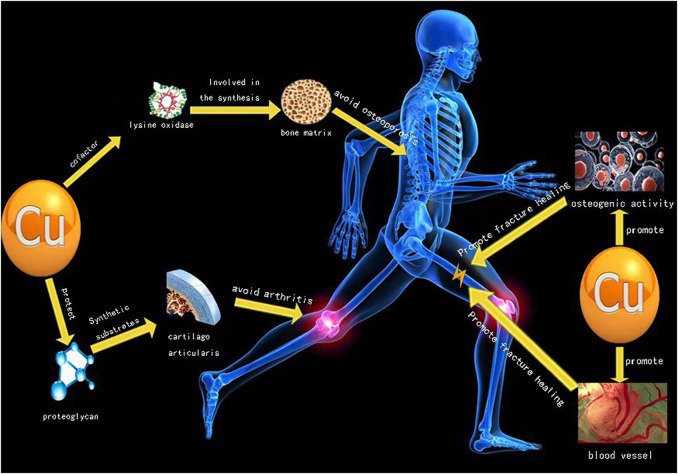
Implications of copper for treating diseases in orthopedics.

Arthritis, fracture and osteoporosis are among the three main diseases in orthopedics, and *Cu* plays an important role in treatment of these three main diseases (Figure 1). The *Cu* content in the human body is between 50 and 120 mg, and nearly two-thirds of *Cu* content is stored in muscles and bones. Liver is the key organ to maintain the content of copper in plasma (Olivares and Uauy, 1996; Turnlund et al., 1998). The content of copper in the body is related to bone deformity, increased osteoporosis, impaired melanin synthesis and immune response during development. When serum copper is deficient, it will increase the frequency of infection, cardiovascular diseases, changes in cholesterol metabolism and other trace element metabolic disorders (Olivares and Uauy, 1996; Turnlund et al., 1998).

Notably, scholars have compared healthy horses with horses suffered from developmental orthopedic disorders, indicating that horses with developmental orthopedic disorders are associated with reduced serum copper levels (Coskun et al., 2016). Furthermore, interference with trace elements, such as a lack of copper, iron, zinc and other metals, has long been regarded as a risk factor for osteoporosis (Hsieh and Navia, 1980; Davey, 1997; Wynchank and Saltman, 1997).

Copper is an essential cofactor of various enzymes involved in the synthesis of various bone matrix components (Lowe et al., 2002). Copper ion is essential for building strong bones *via* regulation of bone metabolism, and studies have shown that post-menopausal women with osteoporosis have significantly lower levels of copper than normal (Mahdavi-Roshan et al., 2015). As a key cofactor of lysine oxidase, copper participates in bone and cartilage metabolism, as a major enzyme involved in collagen cross-linking of cartilage-rich tissues. Animal studies have shown that copper deficiency can lead to bone strength reduction and bone loss, and eventually lead to osteoporosis. One study showed that bone mineral density in children under 3 years of age was positively correlated with age and serum copper levels (Wu et al., 2021b). Either copper deficiency or excessive copper accumulation would be detrimental to bones. Therefore, to maintain a moderate level of serum copper and healthy copper metabolism could facilitate the maintenance of healthy bone remodeling and reduction of the risk of hip fractures. But high levels of serum copper, especially in adult men, can increase the risk of fractures (Qu et al., 2018). Supplementation of trace elements (including copper) can prevent and reduce bone loss (Strause et al., 1994). Patients with thoracic hyperosteogeny (Cox et al., 1983), avascular necrosis of the femoral head (Milachowski, 1988), femoral neck fracture (Conlan et al., 1990), and lumbar osteoporosis had lower copper levels (Howard et al., 1992).

## Involvement of copper in arthritis

Increasing evidence has suggested a disturbance in copper homeostasis and abnormal copper metabolism in rheumatoid disease, a chronic inflammatory disease of the whole joint (Scudder, 1976; Scudder et al., 1978; Rafter, 1987; Sarban et al., 2005). Copper plays an important role in immune response and anti-arthritis. A comprehensive meta-analysis suggests that increased serum level of copper is generally present in rheumatoid arthritis patients. The research history of association between copper and arthritis could be traced back to 40 years ago, Whitehouse et al. detected moderate anti-inflammatory and antiarthritic effects of various copper salts in rats (Whitehouse and Walker, 1978). Cartilage is composed of chondrocytes, water, and cartilage matrix. The extracellular matrix of articular cartilage contains proteoglycans, collagen, and non-collagenous proteins (Kleine and Singh, 1982; Chaminade et al., 1982; Fife and Brandt, 1984). Altered micro-environments in extracellular matrix composition and mechanical properties during arthritis progression are critically involved in arthritis pathology (Maldonado and Nam, 2013; Lin et al., 2020). Thus, targeting of extracellular matrix of cartilage is becoming increasingly attractive for arthritis therapy as modifications to the extracellular matrix (ECM) could be either causal or consequential of arthritis (Schultz, 2019).

Glycoprotein, a 550,000-Da non-collagenous cartilage matrix component, is present in hyaline cartilage and fibrocartilage (Fife et al., 1986). Pasqualicchio et al. (1996) have demonstrated copper supplementation is able to protect articular cartilage against synovial-induced proteoglycan depletion *ex vivo*, which reveals possible mechanisms by which copper exerts its anti-inflammatory and anti-arthritic actions. A series of studies have assessed serum copper levels in patients with rheumatoid arthritis (RA), with conflicting results. This is because inflammatory biomarkers such as interleukin-1 (IL-1), IL-6, and tumor necrosis factor—α (TNF-α) up-regulate the synthesis and secretion of ceruloplasmin in liver cells during rheumatoid arthritis inflammation. When ceruloplasmin is transferred from the liver cell to the serum, the concentration of serum copper increases. Drugs containing copper ions also play an important role in the treatment of gout arthritis. Mubin et al. found that copper oxide nanoparticles could reduce serum levels in rats and treat gout arthritis caused by hyperuricemia (Kiyani et al., 2020). A copper-incorporated bioactive glass-ceramics scaffolds (Cu-BGC) can repair cartilage damage and reduce inflammatory response caused by osteoarthritis. However, a clinical trial suggests that magnetic wrist straps, and also copper bracelets, displayed neither statistically significant, nor clinically meaningful, therapeutic effects upon rheumatoid arthritis. Thus, to find a right therapeutic route on copper-based therapy is essential (Richmond et al., 2013).

To investigate role of copper on osteoclast function. Li and Yu (2007) isolated osteoclasts from the long limb bones of newborn rabbits and cultured them with cu-containing culture medium and deactivated human tooth slices. It was proved that extracellular copper ions could inhibit the absorption of osteoclasts. Previous studies have shown that *Cu* is an important cofactor of many enzymes. It is also an angiogenesis promoting and antibacterial agent. Milkovic et al. (2014) observed that low concentration of *Cu* could improve the viability and growth of human osteoblast like cells. In the treatment of bone defects by bone regeneration, new copper—containing composite materials can enhance osteogenic activity and promote angiogenesis at the early stage of the treatment process. At the same time, the composite can be combined with silicon to complete degradation (Wu et al., 2021a). Based on the research and development of metal manufacturing technology, there are three main methods to prepare cu-containing biomaterials to promote fracture healing *via* 1) supplementation of copper-containing biomaterials; 2) Add copper coating on metal surface; 3) Binding of copper nanoparticles with other functional bio-metals (Wang et al., 2021).

## Copper involvement in cellular functions of mesenchymal stem cells

Mesenchymal stem cells are one of the most intensively studies adult stem cells. Bone marrow mesenchymal stem cells can reduce the proinflammatory potential of dendritic cells by inhibiting the production of tumor necrosis factor during innate immunity. In addition, incubation of plasmacytoid dendritic cells with mesenchymal stem cells up-regulated the production of the anti-inflammatory cytokine IL-10 (Aggarwal and Pittenger, 2005). Therefore, the combination therapy of mesenchymal stem cells on dendritic cells and plasmacytoid dendritic cells may be translated into effective anti-inflammatory and immunomodulation-associated therapeutics (Aggarwal and Pittenger, 2005; Uccelli et al., 2008). Mesenchymal stem cells have been proved to inhibit respiratory burst and delay the spontaneous apoptosis of dormant and activated neutrophils through IL-6-relevant mechanism (Raffaghello et al., 2007). Therefore, mesenchymal stem cells play an important role in the resting preservation of neutrophils and avoiding respiratory burst (Craddock et al., 1960; Uccelli et al., 2008).

Hypoxia inducible factors (HIFs) play a central role for cellular adaptation to low oxygen microenvironments through regulation of diverse downstream genes, which is also closely associated with the energy metabolism and immune alterations (Jaakkola et al., 2001). During inflammatory response, infected and inflamed tissues are usually hypoxic, and HIFs are conducive to the adaptation and normal functioning of various types of immune cells (Finlay et al., 2012). HIF-1 consists of two subunits: HIF-1α and HIF-1β. Under anoxic condition, HIF-1α abscond from the disassemble pathway, accumulate collect in the cytosol, and is translocated into the nucleus, where it dimerizes with HIF-1β, interacts with cofactors to assemble the HIF-1 transcriptional complex, and binds to the hypoxia-responsive element sites of its target genes, leading to transactivation of target genes expression. About 300 genes are regulated by HIF-1α (Fedele et al., 2002; Greijer et al., 2005). Hypoxia-inducible factor (HIF)-1-dependent signalling pathways regulating bone healing. Once bone injury or hypoxia happens, HIF-1a activation and stabilization occur. Vascular endothelial growth factor VEGF, stromal cell-derived factor-1, and CXC chemokine receptor CXCR 4 are directly positively regulated by HIF-1a. Increased expression of VEGF, SDF-1, and CXCR4 stimulates mesenchymal stem cell (MSC) homing (Lin et al., 2017). Remarkably, copper is essential for transactivation of HIF-1α for regulation of various of target gene expression (Martin et al., 2005). Additionally, Burghardt et al. found that copper in the concentration range of 0.1 mM could promote the proliferation of MSC and the osteogenic differentiation of MSCs. He also observed that copper could increase the activity of alkaline phosphatase in MSCs, and the expression of osteogenic markers such as type I collagen, o osteocalcin (OCN), and osteopontin (OPN) (Burghardt et al., 2015). The results of Li et al. (2019) suggest that sodium copper chlorophyllin can not only promote the proliferation and differentiation of bone marrow mesenchymal stem cells in mice with aplastic anemia, but also improve their immune regulation ability.

## Conclusion

This review summarizes the effects of copper ion on immune response, arthritis, osteoporosis and fracture, and expounds the application progress of copper ion in biomedicine and copper-containing materials. As an important part of immune response, copper ions are released from ceruloplasmin at the stage of inflammatory reaction, resulting in increased serum copper. Copper ions can also achieve the effect of fighting pathogenic bacteria through the “contact killing” property.

The application of cu-containing nanomaterials and cu-containing coatings in promoting fracture healing and antibacterial research has attracted increasing attention of scholars. The application of cu-containing drugs and materials can avoid the issues of high cost and easy loss of biological activity, and can strongly stimulate the expression of related genes, with better clinical safety, thus inducing bone tissue repair and regeneration to complement each other. In conclusion, copper will provide a safe, efficient, and innovative activity platform for the biomedical field.
